# Low risk management intervention: Limited impact of remedial tillage on net ecosystem carbon balance at a commercial Miscanthus plantation

**DOI:** 10.1111/gcbb.13114

**Published:** 2023-12-08

**Authors:** R. L. Rowe, H. M. Cooper, A. Hastings, A. Mabey, A. M. Keith, N. P. McNamara, R. Morrison

**Affiliations:** ^1^ UK Centre for Ecology and Hydrology Lancaster Environment Centre Lancaster UK; ^2^ UK Centre for Ecology and Hydrology Wallingford UK; ^3^ School of Biological Sciences, Institute of Biological and Environmental Sciences University of Aberdeen Aberdeen UK; ^4^ Biological Sciences University of Southampton Southampton UK; ^5^ School of Ocean and Earth Science, National Oceanography Centre Southampton University of Southampton Southampton UK; ^6^ Present address: BeZero Carbon Ltd. London UK

**Keywords:** crop management, eddy covariance, Miscanthus, soil carbon

## Abstract

Perennial bioenergy crops are a key tool in decarbonizing global energy systems, but to ensure the efficient use of land resources, it is essential that yields and crop longevity are maximized. Remedial shallow surface tillage is being explored in commercial Miscanthus plantations as an approach to reinvigorate older crops and to rectify poor establishment, improving yields. There are posited links, however, between tillage and losses in soil carbon (C) via increased ecosystem C fluxes to the atmosphere. As Miscanthus is utilized as an energy crop, changes in field C fluxes need to be assessed as part of the C balance of the crop. Here, for the first time, we quantify the C impacts of remedial tillage at a mature commercial Miscanthus plantation in Lincolnshire, United Kingdom. Net ecosystem C production based on eddy covariance flux observations and exported yield totalled 12.16 Mg C ha^−1^ over the 4.6 year period after tillage, showing the site functioned as a net sink for atmospheric carbon dioxide (CO_2_). There was no indication of negative tillage induced impacts on soil C stocks, with no difference 3 years post tillage in the surface (0–30 cm) or deep (0–70 cm) soil C stocks between the tilled Miscanthus field and an adjacent paired untilled Miscanthus field. Comparison to historic samples showed surface soil C stocks increased by 11.16 ± 3.91 Mg C ha^−1^ between pre (October 2011) and post tillage sampling (November 2016). Within the period of the study, however, the tillage did not result in the increased yields necessary to “pay back” the tillage induced yield loss. Rather the crop was effectively re‐established, with progressive yield increases over the study period, mirroring expectations of newly planted sites. The overall impacts of remedial tillage will depend therefore, on the longer‐term impacts on crop longevity and yields.

## Graphical abstract

Remedial surface tillage has been proposed to improve Miscanthus yields by redistributing rhizomes and reducing canopy gaps, however, tillage is often associated with soil carbon losses. We studied remedial tillage in a commercial Miscanthus plantation and observed a temporary reduction in yield but no impact on soil carbon sequestration compared to a paired untilled site. Whole field eddy covariance measurements over a 5 year period confirmed that the plantation was a carbon sink (20.68 Mg C ha^−1^). Yield modelling estimated 12.16 Mg C ha^−1^ of this carbon remained within the plantation whilst 8.52 Mg C ha^−1^ was exported as harvested material.

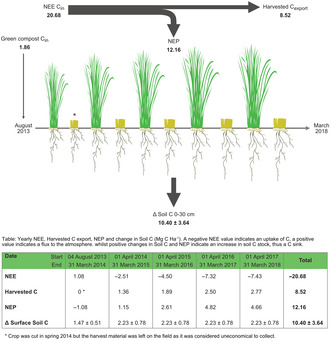

## 1 INTRODUCTION

*Miscanthus* × *giganteus* Greef et Deu (hereafter abbreviated to Miscanthus) is grown as a bioenergy feedstock in temperate climates such as Europe and the USA (Anderson et al., 2011). Able to grow on marginal lands and with minimal fertilizer requirements, this long lived (>15 years) tall rhizomatous C_4_ grass, is seen as an ideal candidate to meet the growing demand for sustainable biomass feedstock (Committee on Climate Change, 2019; McCalmont, McNamara, et al., 2017; Richter et al., 2016). Miscanthus, however, is currently a marginal value, high volume crop and therefore the financial returns to growers and the profitability of Miscanthus bioenergy systems require high yields to be realized over time (Panoutsou & Chiaramonti, 2020). Maximizing yields also ensures the efficient use of land helping to reduce concerns over potential land use conflicts and indirect land use change (Calvert & Mabee, 2015; Pogson et al., 2013; Trainor et al., 2016).

In the UK, open patches within the cropped area of commercial Miscanthus plantations have been identified as causing yield losses of up to 37% compared to potential yields (Richter et al., 2016). Caused by aging and/or poor establishment (Richter et al., 2016; Zimmermann et al., 2014), these open patches reduce land use efficiency and can significantly impact financial returns, with Zimmermann et al. (2014) reporting that in a study of six commercial farms in Ireland, open areas covered an average of 13.7% of the field area, ranging from 7.98% to 19.31%. This significantly impacted payback time on the initial investment in the worst cases could potentially reduce gross margins by over 50% over the lifetime of the crop. Ongoing improvements in planting technologies are reducing cases of poor establishment, but cases can still occur, and the gaps caused by aging crops will increase as the first commercial planting of these crops in the early 2000s mature (Shepherd et al., 2020). Replanting these crops is an option but it carries a significant cost and, recognizing this, remedial tillage, is being trialled by crop agronomists as a lower cost method to rectify open patches (Terravesta, 2018). This method involves disc harrowing (shallow tillage utilizing metal disks) to divide and redistribute the propagule rhizomes, followed by rolling (with large, weighted rollers) to firm the soil around the newly divided rhizomes (Terravesta, 2018). This stimulates new vigorous growth in older crops and evens out rhizome distribution across open patches (Terravesta, 2018). There are, however, risks of unintended impacts on the carbon (C) balance of the crop.

Tillage in conventional agricultural systems has been widely associated with increased soil carbon dioxide (CO_2_) emissions and losses of soil C (Chenu et al., 2019; van Groenigen et al., 2011), and tillage associated with Miscanthus crop removal is thought to contribute to reported increases in soil greenhouse gas (GHG) fluxes (Drewer et al., 2016; Dufossé et al., 2014; McCalmont et al., 2018) and soil C losses during crop removal (Rowe et al., 2020). Whilst both annual tillage in agricultural systems and tillage associated with bioenergy crop removal are more intensive processes than remedial tillage, any losses of soil C in bioenergy systems may negatively impact the overall C balance of the crop (Rowe et al., 2020). Miscanthus yields are also expected to be temporarily reduced by remedial tillage as rhizomes will need to establish root networks (Terravesta, 2018), thereby further impacting the C balance of the crop and reducing the capacity of the land to offset fossil fuel use. To understand if these negative impacts can be potentially offset by the expected gains in longer‐term yields or through extending the crop lifetime, it is critical to assess the scale, duration and magnitude of the impacts of remedial tillage on yields and soil C fluxes and stocks, however these remain unquantified.

Here, exploiting a unique opportunity where a remedial tillage trial was undertaken as part of a commercial operation on one of two adjacent Miscanthus fields in Lincolnshire UK, we address this knowledge gap. We combine eddy covariance (EC) measurements of CO_2_ fluxes within the tilled Miscanthus field, providing high resolution values for net ecosystem CO_2_ exchange (NEE) for the 4.6 years following the tillage event, with soil C stock sampling (0–70 cm) of the tilled and adjacent untilled fields, and historical pre‐tillage soil samples, to assess changes in C stocks. We also recorded crop yield using harvest statistics at the farm level in combination with field‐specific modeled estimates of crop yield for the tilled field. Using this data our objectives were: (i) to quantify the NEE of the Miscanthus plantation following tillage using EC; (ii) to analyze temporal trends in C exchange during the years following remedial tillage; (iii) to quantify impacts on soil C stocks of the remedial tillage; and, (iv) to quantify the major C flow following tillage using NEE, yield estimates and rates of change in soil C stock.

## 2 METHODS

### 2.1 Site description

The site is a commercial farm approximately 10 km north of the City of Lincoln in Eastern England, UK. The climate is temperate maritime (Beck et al., 2018) characterized by cool summers, mild winters and a long thermal growth season (Table 1). Mean annual air temperature and precipitation are 9.8 ± 0.7°C and 614 ± 93.5 mm year^−1^, respectively (Table 1). Soils are stagnogley, seasonally waterlogged fine loams and clays overlying Charnworth Mudstone or chalk till (Drewer et al., 2012).

**TABLE 1:** Site characteristics of the Miscanthus study site in Lincolnshire, UK.

Site characteristic	Details
Northing	53.3°
Easting	−0.58°
Elevation	18 m amsl
Climate	
Mean air temperature (1981–2010)	9.8 ± 0.7°C
Thermal season	April to October
Coldest month	January (4.0 ± 1.6°C)
Warmest month	July (16.9 ± 1.2°C)
Mean annual precipitation (1981–2010)	614 ± 93.5 mm year^−1^
Prevailing wind	Southwest
Soils	
Soil	Stagnogley soil, overlaying mudstone or chalk till, boarding both Beccles 1 and Wickham 1 soil associations
Soil texture	49% sand, 36% silt, 15% clay
Soil pH	pH 6.8–7.3
Soil bulk density	1.46 g cm^−3^
Soil carbon concentration (0–30 cm)	1.9%
Soil nitrogen concentration (0–30 cm)	0.2%
Land use and management	
Field size	
Tilled Miscanthus field (Mis A)	11 ha
Untilled Miscanthus (Mis B)	11 ha
Arable control	7.5 ha
Miscanthus fields	
Variety	*Miscanthus* × *giganteus* Greef et Deu
Planting density	10,000 rhizomes ha^−1^
Establishment year	2006
Establishment technique	Rhizomes
Previous land use	Winter wheat (main), oilseed rape (break)
Tillage of Mis A	Late spring 2013
Arable fields	
Cereal rotations	Winter wheat (main), oilseed rape (break)

*Note*: Climate data from RAF Waddington (53°10′30″ N; 0°31′15.6′′ W 68 m asl) 17 km from the field site, data supplied by the Met Office. Soils data are from Cranfield University (2018) (Drewer et al., 2012; Robertson, Whitaker, et al., 2017). Land use information was provided by the land owner.

#### 2.1.1 Miscanthus management

**FIGURE 1:**
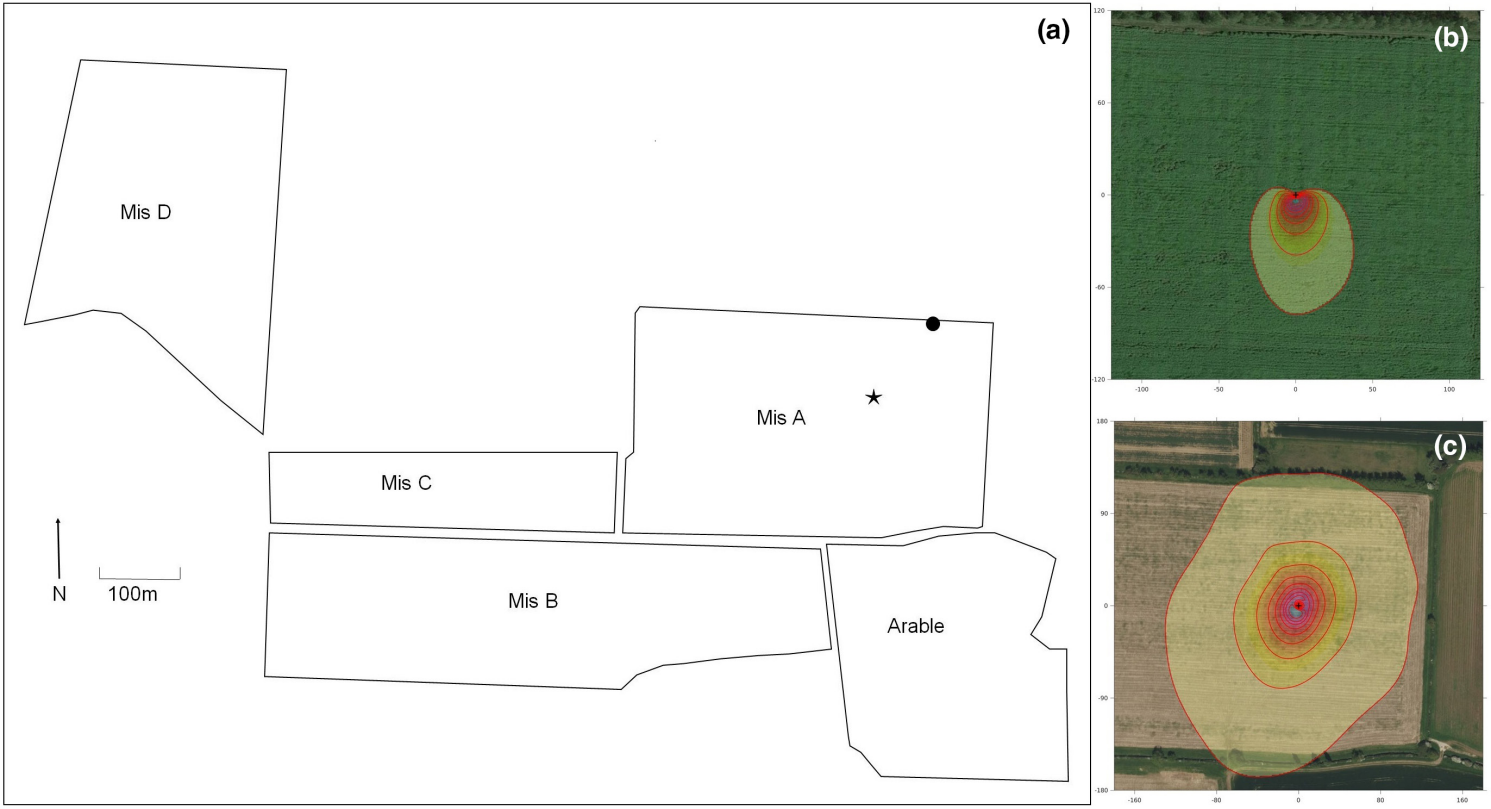
Site map, showing location of the field which underwent remedial tillage in 2013 (Mis A), the remaining untilled Miscanthus fields, and the adjacent arable field (a). Composite yield data were provided by the company that manages the Miscanthus plantations, which included the additional field Mis C, and Mis D. The location of the eddy covariance (EC) tower within the Mis A field (black dot) and the location of the EC mast (star). Minimum (2013) and maximum (2017) extent of the EC footprint climatology are shown [panel (b) and (c), respectively]. Contour lines show contribution of the footprint to the flux measurement in steps of 10%, Flux tower is show as red dot. Footprints were generated via the Flux Footprint Prediction (FFP) online data processing tool (Kljun et al., 2015). For footprints for each year 2013–2017, see Supporting Information (Figure S2).

The farm cultivates four fields of mature commercial Miscanthus, established in spring 2006 on former arable land (main crops winter wheat, break crop oilseed rape), together with a remaining arable field which has continued under the original crop rotation (Figure 1). The Miscanthus fields were established from rhizomes planted at a density of 10,000 rhizomes ha^−1^ using a modified semiautomatic potato planter. Annual spring biomass harvests commenced from March 2008. Farm Miscanthus yields were previously reported at 6.95, 10.28, 6.24, 7.58 and 6.87 dry Mg ha^−1^ for years 2009–2013, correspondingly (Robertson, Whitaker, et al., 2017). In response to progressive yield decline, and open areas within the crop, one of the Miscanthus fields (Mis A, Figure 1) was selected for a remedial tillage trial, to be extended to other fields if successful (Landowner, 2013, personal communication). No assessment of the open areas was conducted at the time but assessment of historical satellite images show both large and small open areas throughout the field in 2008 (Figure S1). Open areas and areas with poor establishment were estimated to cover 25% of the total crop areas. Post tillage images from 2020 shows post tillage these gaps are no longer present (Figure S1). Remedial tillage occurred post‐harvest in late spring 2013, cultivation included two passes with a disk harrow (Simba‐Solo™) to divide the rhizomes, followed by two passes with a set of Cambridge rollers, to ensure good contact between the freshly divided rhizome and the soil (Terravesta, 2018). Green wood waste compost (46.6% C, 1.2% N, C:N = 38) was subsequently applied at a rate of 4 Mg ha^−1^ during July 2013 to all the Miscanthus fields (Keane et al., 2019). The Miscanthus fields received further amendment with 500 Kg ha^−1^ Fibrophos (Fibrphos Ltd., Suffolk, UK) on 25th April 2014, and were sprayed with Aquaclean™ plant promoting bacteria (Blue Plant Labs, NJ, USA) in spring 2016.

### 2.2 Eddy covariance

Turbulent flux (hereafter fluxes) of NEE (μmol CO_2_ m^−2^ s^−1^) and latent and sensible heat (LE and *H*, respectively; W m^−2^) were measured in the tilled Miscanthus field (Figure 1) between 4th August 2013 and 25th November 2017 using an open‐path EC system. Full details of the EC set up and data handing are provided in the open‐access dataset that accompanies this manuscript (Morrison et al., 2019) and only summary details are provided here.

#### 2.2.1 Instrumentation

The EC system comprised a Solent R3 sonic anemometer‐thermometer (Gill Instruments, Lymington, UK) and an LI‐7500 infrared gas analyser (LI‐COR Biosciences, Lincoln, NE, USA). Raw EC data were logged at 20 Hz using a CR3000 Micrologger (Campbell Scientific, Inc., Logan, UT, USA). EC instruments were mounted on a pneumatic mast (Clarke Masts, Binstead, UK) that was extended to maintain the height of the EC sensors at a minimum of 2 m above the Miscanthus canopy. The EC system was installed on an extendible pneumatic mast (Clarke Masts Ltd., Binstead, UK) at a central position within the plantation, with a slight bias towards the NE corner to maximize the available fetch from the prevailing SW direction, and partially as this position was preferable to the land owner (Figure 1).

Ancillary meteorological and soil physics data were measured from a scaffold tower at the northern edge of the field. Measurements included: the net radiation (*R*_net_; W m^−2^; CNR1; Kipp & Zonen, Delft, The Netherlands) and its incoming and outgoing short‐ and long‐wave components; soil heat fluxes at 0.03 m (*G*; W m^−2^; HFP01; Hukesflux, Delft, The Netherlands; *n* = 2); air temperature (*T*_a_; °C) and relative humidity at 2 m (RH; %; HSC2; Rotronic Instruments Ltd., Crawley, UK); precipitation (*P*; mm [0.5] h^−1^; Didcot Instruments Ltd., UK); and the volumetric water content of the upper 0.3 m soil profile using a pair of vertically inserted CS616 water content reflectometers (VWC; m^3^ m^−3^; Campbell Scientific, Inc.; *n* = 2). Soil temperature (*T*_s_, °C) was measured at 0.05 m below the surface at two locations using PT107 soil thermocouples (Campbell Scientific, Inc.).

#### 2.2.2 Flux data handling

Fluxes were calculated using EddyPRO® Flux Calculation Software Version 6.1 (LI‐COR Biosciences; Fratini & Mauder, 2014), using default settings for all procedures and corrections to compute fluxes from raw EC data using block averages and 30‐min flux averaging periods (see Morrison et al., 2019 for details). Positive flux represent net C emission to the atmosphere and negatives denote the reverse.

**FIGURE 2:**
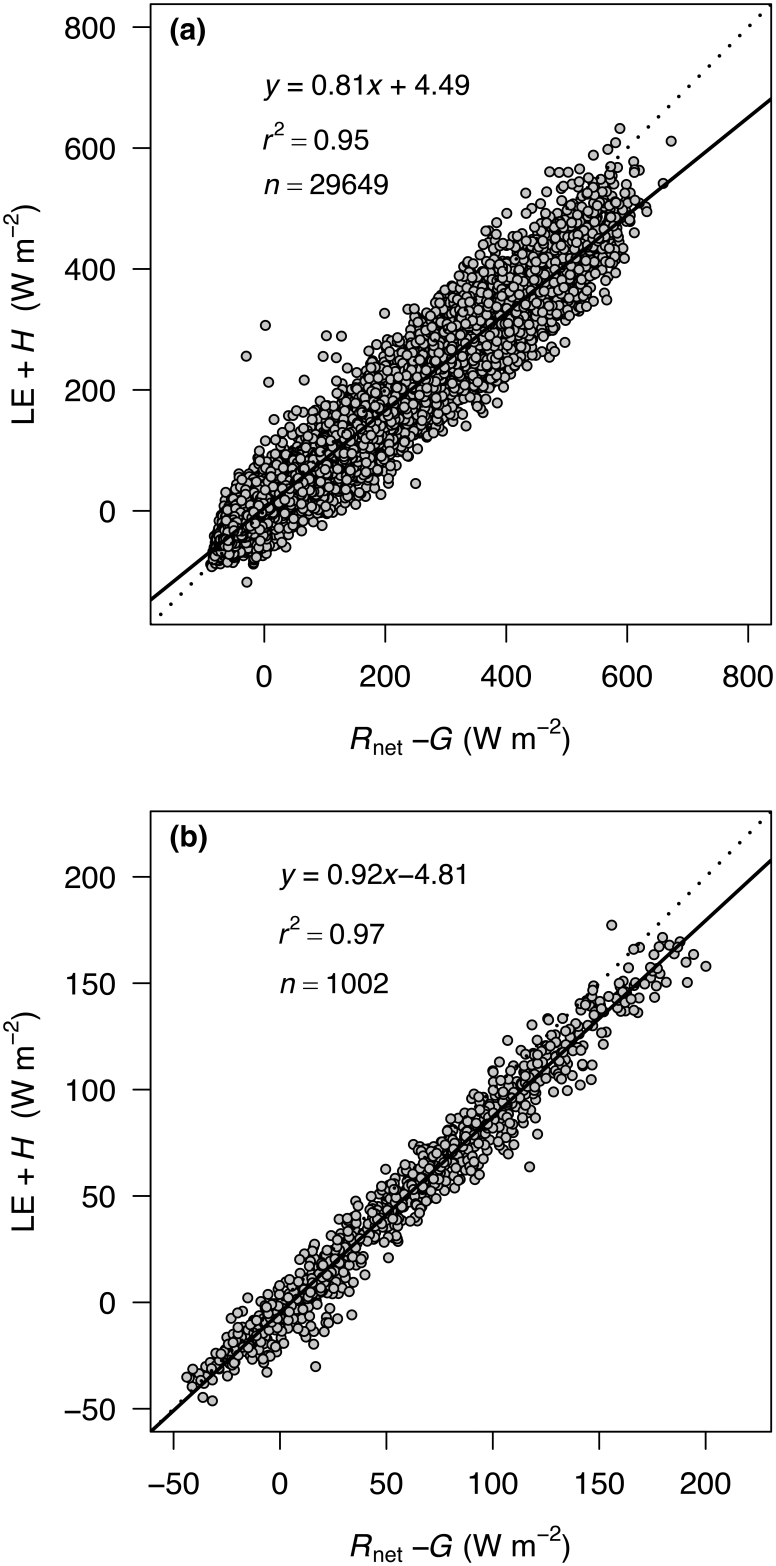
Energy balance closure at a Miscanthus plantation in Lincolnshire, UK, evaluated using (a) 30 min flux observations and (b) daily averages of the energy balance terms.

Quality control (QC) of 30 min flux data included: (i) removal of statistical outliers (Papale et al., 2006); (ii) rejection of data when the AGC parameter of the LI‐7500 was 10% above its baseline (Ruppert et al., 2006); (iii) when stationarity test results deviated by more than 100%; and (iv) when fluxes were outside realistic ranges for the site. NEE data were excluded when they were negative at night (defined as *R*_g_ < 20 W m^−2^) and below a fiction velocity (u*) threshold of 0.16 m s^−1^ estimated according to Reichstein et al. (2016). Fluxes were considered spatially representative when a two‐dimensional footprint model predictions indicated that >80% of the flux originated within the Miscanthus field (Kljun et al., 2015). Missing data due to the application of QC resulted in the capture of data during 63% of the measurement period. Similar to other EC studies, data completeness was higher during daytime (70%) that at night (56%). Gap‐filling and the partitioning of NEE into estimates of gross primary production (GPP) and total ecosystem respiration (TER) were performed using the REddyProc Package (Reichstein et al., 2016) for R (R Core Team, 2019). Energy balance closure was 81% and 92% when evaluated using 30 min observations and daily means of the energy balance terms (Figure 2), respectively, falling within the 55%–99% range for EC sites globally (Leuning et al., 2012; Stoy et al., 2013; Wilson et al., 2002).

### 2.3 Vegetation measurements and yields

With the exception of 2014 when destructive samples were used to estimate crops yields (Keane et al., 2019), yield data were provided by the company that manages the Miscanthus plantation, however, only composite yields incorporating all four fields were available. In addition, in 2016 Miscanthus Field D was not harvested due to extremely wet conditions and instead sub‐soiling was conducted to improve drainage (Landowner, personal communication). Combined, these factors made isolating the impact of tillage on yields difficult and highlights challenges of making real world observations under commercial operations. Thus, in addition to site based yield measurements, modeled yields for the tilled field were produced using the MiscanFor model, version MiscaForSPI_24, a process based crop growth model described in Hasting et al. (2009). MiscaForSPI_24 was parameterized for the site using daily meteorological data, obtained from RAF Scampton, 4 km distance, supplemented by measurements at the site. Soil parameters required by the model were calculated from soil texture, bulk density and % C derived from soil sampling (see below). Field capacity, wilting point and soil organic carbon (SOC) of the soil were derived using the Campbell method described in Hastings et al. (2014). The model was parameterized by matching the commercial yield to the model output in the period before tillage in 2013, which resulted in a maximum radiation use efficiency factor of 1.96. Tillage was simulated in the model by considering that chopping the crops rhizomes was the same as replanting the crop and to model the crop's recovery, the model was run with the expected sigmoidal new crop establishment curve for *Miscanthus* × *giganteus* (Shepherd et al., 2020). The model calculates the above ground biomass (AGB), which is reduced from its peak yield by 33% to account for leaf drop and nutrient repartition to the rhizome to calculate the harvested yield. To match the AGB to the EC Carbon exchange the modelled biomass was considered to be 44% carbon and the annual below ground C increment was considered to be the same as the AGB C (Martani et al., 2021). Daily increments in biomass C calculated by the model were summed to give monthly increments to compare both daily and monthly values to the EC data. Modelled yields agreed well with the whole site yields, as well as yield estimates based on NPP derived from flux tower measurements (see Figures S3 and S4).

Both modelled and measured yield were converted to C exported from the site at harvest (C_export,_ g C m^−2^) based on the dry weight of the harvested biomass using a conversion factor of 0.44 (McCalmont, McNamara, et al., 2017).

### 2.4 Net ecosystem production

Net ecosystem production (NEP) was calculated following Equation (1), giving the net change in the field C balance based on the NEE from the EC and the estimates of C_export_ from the modelled crop yields, and converted to a positive value where balance would result in an up take of C from the atmosphere. NEP was calculated for five harvest cycles between 4 August 2013 to harvest in 31 March 2018. Due to vandalism of the EC system, measurement ceased on 25 November 2017. In the absence of further flux or meteorological data acquisition at the site, winter fluxes for December 2017 to the harvest in March 2018 were estimated as the mean of monthly C fluxes measured during the winter months of all previous years. It is acknowledged that this approach adds additional uncertainty to the estimate of NEP for the final year of data collection, however, winter fluxes at this location are significantly lower, than during the crop's growth period. Mean monthly winter (December–March) rainfall and temperatures in 2017 (55.34 ± 16.34 mm, 4.33 ± 1.0°C respectively), were also within the standard deviations of the previous four growth periods of 43.00 ± 17.26 mm and 5.79 ± 1.58°C. Therefore, the benefit of estimating the complete 2017 growth period (March–March) was felt to be justified.

(1) $$\mathrm{NEP}=-1\left(\mathrm{NEE}-C_{\text{export}}\right),$$

where all variables were defined above. Uncertainty in time integrated sums of NEE was estimated according to (Levy & Gray, 2015), using standard error propagation techniques based on standard deviations estimated for observed (Finkelstein & Sims, 2001) and gap‐filled (Reichstein et al., 2016) flux. Fluvial C fluxes were assumed to be negligible as were other GHG (CH_4_ and nitrous oxide, N_2_O) emissions based on previous chamber studies, which indicated these emissions and emissions/removals of methane, contribute little to the C and GHG balance of the plantation beyond the early establishment phase (Drewer et al., 2012; Keane et al., 2019; Robertson, Whitaker, et al., 2017).

### 2.5 Soil sampling

The soil sampling had two aims, to quantify the impacts on soil C stocks of the remedial tillage, and to calculate rates of change in SOC stock. The latter being required to relate changes in soil C stocks to the C fluxes measured during the period during which the EC flux measurements were available.

#### 2.5.1 The impacts of remedial tillage

The impacts of remedial tillage were explored using a paired site, space for time approach (De Palma et al., 2018), comparing soil C stocks in the tilled Miscanthus field (Mis A) with the adjacent untilled Miscanthus field B (Mis B) (Figure 1). Miscanthus field B being selected as, of the fields available, it provided the closest match to Mis A in terms of size, location, management and condition of the crop. This is critical for a paired site approach where differences are assumed to be caused by the management intervention of interest, in this case tillage (De Palma et al., 2018).

Soil sampling was conducted on 9th of November 2016, 3.5 years after the tillage event, and consisted of a combination of surfaces soil samples (0–30 cm) and deep samples (0–1 m). The sampling protocol followed (Rowe et al., 2020) and is given in detail in supplementary material. Briefly, five sampling plots per field were randomly selected (Figure S5) and three soil cores taken from each sampling point, using a split‐tube soil sampler (Eijkelkamp Agrisearch Equipment BV, Giesbeek, The Netherlands) to a depth of 30 cm (see Figure S5 for details). Cores were sectioned into 10 cm increments (0–10, 10–20 and 20–30 cm) in the field. At three randomly selected locations, the 30 cm coring was extended to 1 m by using a window sampler system (Eijkelkamp Agrisearch Equipment BV). The window sampling system seals the cores within a plastic sheath during sampling and the cores were transported in one section to the laboratory for processing.

##### 2.5.1.1 Laboratory processing

On return to the laboratory, visual inspection of the 1 m cores revealed that within the untilled field there was a greater influence of the parent material in the lower section (>0.70 m) than in the tilled field. Thus, while all samples were processed as outlined below, statistical analysis and comparison between these fields were limited to the upper 0–0.70 m.

Meter cores were divided into 10 cm increments and each core section from both the window sampler (0–1 m) and split core sample (0–30 cm) were processed following methods in Rowe et al. (2016). Briefly, core sections were dried and sieved to 2 mm, with moisture loss and volume and mass of stones and roots recorded. The oven dry soil mass of each core section was calculated using values of moisture‐loss, and stone and root volume using the following methods used in the GB Countryside Survey (Reynolds et al., 2013). An oven‐dried subsample of soil (~20 g) was then ground in a ball mill (Fritsch Planetary Mill) and 100–200 mg samples were weighed into silver cups (5076; Elemental Microanalysis, Okehampton, UK), higher weights used for sub‐soil where C content is lower. Samples were then treated with hydrochloric acid (HCl) following methods in Rowe et al. (2020) to remove inorganic C. Samples were then wrapped in a second tin cup to aid combustion before being assessed for C concentration in an elemental analyser (Leco Truspec CN, Milan, Italy).

##### 2.5.1.2 Soil carbon stock calculations

The soil C concentration and soil mass of the core sections were used to calculate soil C stock on an equivalent soil mass (ESM) approach for each core (Gifford & Roderick, 2003). This was done for the two depths, using a reference dry soil mass of 4000 and 10,390 Mg ha^−1^ for the 0–30 and 0–70 cm soil depths, respectively, following Equation (2). Reference soil masses being based on the median soil mass across all cores for each depth (Gifford & Roderick, 2003).

(2) $${\mathrm{SC}}_{\mathrm{ESM}}={\mathrm{SC}}_{\text{upper}}+\left({\text{Conc}}_{\text{Lower}}\;\left(M_{\mathrm{ref}}-M_{\text{upper}}\right)\right),$$

SC_ESM_ is the soil C stock based on the selected ESM (Mg C ha^−1^), SC_upper_ is the C stock (Mg C ha^−1^) of the upper soil section, Conc_Lower_ is the C concentration of the lower layer (%C), *M*_ref_ is the reference mass selected (Mg ha^−1^) and *M*_upper_ is the soil mass of the upper core sections (Mg ha^−1^). Summed values of the 0–10 cm and 10–20 cm were used as the upper section with 20–30 cm as the lower section. In the 0–70 cm depth the upper section defined as the 0–60 cm layer and the lower section being the 60–70 cm layer.

#### 2.5.2 Rate of change

To calculate the rates of change in surface soil C stocks (0–30 cm), ESM soil C values from the tilled Miscanthus field (Mis A) were compared to historical samples taken from this field on 21st October 2011, 1.5 years prior to the tillage (Rowe et al., 2016). Sampling and laboratory methods were consistent between the two sampling dates, based on the same sampling locations, coring equipment and analytical methods (Rowe et al., 2016). There were minor differences in the method used to remove inorganic C from the samples, and in the depth increments into which cores were divided, with cores in 2011 divided into 15 cm increments compared to 10 cm (Rowe et al., 2016). To address methodology differences in the removal of the inorganic C, archived balled milled, but not HCl treated, samples from 2011 were accessed, and reprocessed alongside the 2016 samples, ensuring consistent comparisons were made. Differences in depth increments will have limited impact on the results as comparison of soil C stocks were made on an ESM approach following Equations (2) and (3):

(3) $${\text{ΔSOC}}_{\mathrm{pre}-\text{post tillage}}=\left[{\mathrm{SOC}}_{\text{post till}}-{\mathrm{SOC}}_{\mathrm{pre}\;\text{till}}\right]/\left[t_{\text{post till}}-t_{\mathrm{pre}\;\text{till}}\right].$$

The pre‐post tillage rate of change is given by dividing the difference in mean surface soil C stocks (SOC) based on ESM, between the post‐tillage (2016) and pre‐tillage (2011) Mis A field samples, by the time (t) between the sampling dates (5 years).

These historical data also included samples taken from the adjacent arable field (Rowe et al., 2016). These samples were also included in the reanalysis to allow rates of change in soil C stock to be calculated for the whole lifetime of the crop, with the arable crop used as a space for time proxy for the pre‐Miscanthus land use (Equation 4):

(4) $${\text{ΔSOC}}_{\text{crop life time}}=\left[{\mathrm{SOC}}_{\text{post till}}-{\mathrm{SOC}}_{\text{Arable}}\right]/\mathrm{Age}\;\text{of the crop}.$$

The crop lifetime rate of change is given by dividing the difference in SOC stock between the post tillage samples in 2016 and the arable field by the age of the Miscanthus crop at the time of sampling (10 years).

#### 2.5.3 Ecosystem C dynamics

To explore the ecosystem carbon dynamics, NEP giving the net change in the field C balance was examined in conjunction with changes in soil C stock. As the time periods covered by the soil C sampling (2011–2016) and the EC measurements (2013–2017) do not align, changes in soil carbon stock over the EC measurement period were estimated based on the pre‐post tillage rate of change. This was done by multiplying the pre‐post tillage yearly rate of change by the time‐period of interest (4.6 years) from post tillage in 2013 till harvested in March 2018 to give an estimated change in soil C stocks over this period. This approach must be interpreted with due caution as it assumes a linear and consistent rate of change in soil C stock, something which may not be the case in field conditions (Qin et al., 2016). However, there is considerable overlap between the time‐period covered by each sampling method; thus, this approach was considered to provide a reasonable approach for the purposes of comparing soil C changes to the EC measurements.

#### 2.5.4 Statistical analysis

Impacts on soil C stocks were compared on an ESM basis using mixed effect models with the *nlme* package in the R statistical program (Pinheiro et al., 2013). For the assessment of impacts on surface soils (0–30 cm) data from all fields and sampling years were included. Management (tilled, untilled, pre‐tilled and arable) was included as the only fixed factor, sampling date was excluded as this cannot be separated from management, however to account for the repeated sampling of the Mis A field, the term field (Mis A, Mis B, Arable) was included as a random factor, with plot nested within this. The significance of these models was examined using a likelihood ratio test between a null model, including only random terms, and the chosen models with fixed terms. With significant interactions tested using Tukey pairwise post‐hoc test. For the assessment of the 0–70 cm soil C stocks, data from only the tilled (Mis A) and the untilled (Mis B) fields were included in the analysis. The linear model was the same as used for the surface soil stock but with the random “field” term removed as it was no longer required.

## 3 RESULTS

### 3.1 Monthly dynamics in C fluxes

**FIGURE 3:**
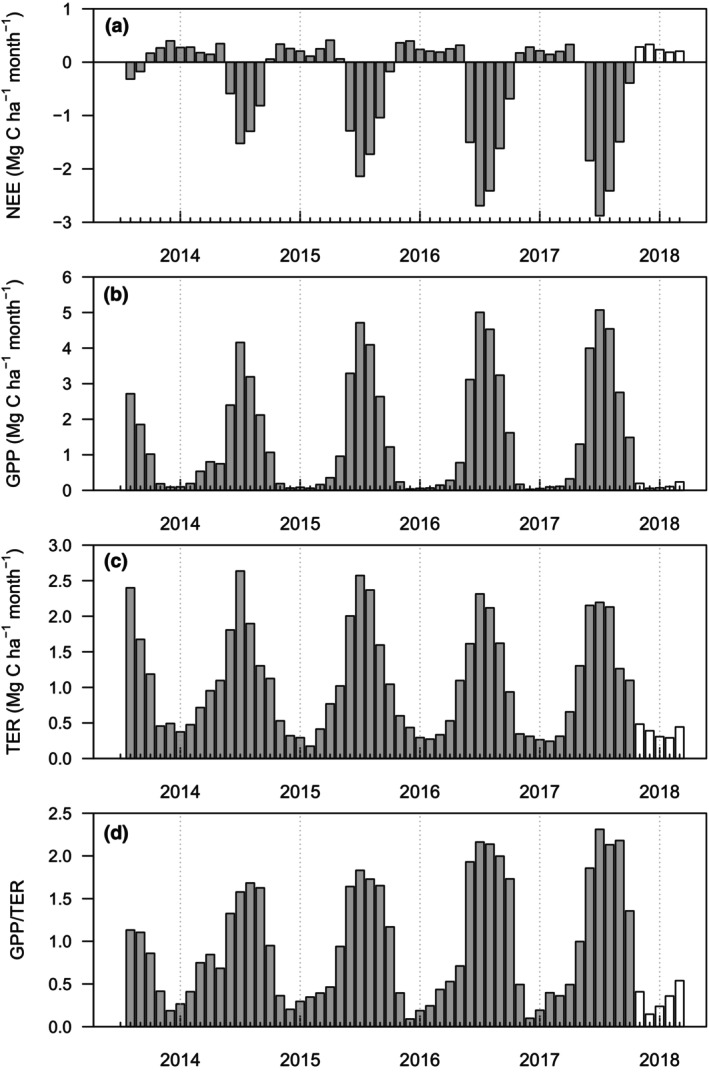
Monthly values of (a) total monthly net ecosystem exchange (NEE), (b) total gross primary production (GPP), (c) total ecosystem respiration (TER) and (d) the ratio of GPP to TER for a commercial Miscanthus plantation in Lincolnshire. Data are for the period August 2013 to November 2017. The white bars for December 2017 to March 2018 are the averages of the monthly values observed during previous years.

Despite clear differences in the magnitude of ecosystem CO_2_ fluxes, the seasonal dynamics of ecosystem CO_2_ fluxes where broadly similar in all year with net (NEE) C emission over winter and C uptake during spring and summer (Figure 3). This reflects the pattern of crop growth (GPP) which begins in early spring, peaks in July before slowly declining into the autumn (Figure 3).

The magnitude of GPP showed a progressive increase with time since tillage, with annual totals ranging from 8.78 Mg C ha^−1^ year^−1^ post‐tillage to a maximum of 19.58 Mg C ha^−1^ year^−1^ for the 2017 growth year. In contrast to GPP, TER values, excluding the initial two‐year post tillage, showed a declining trend with time since tillage. For the available full annual periods, TER ranged from 13.31 Mg C ha^−1^ year^−1^ (2015 growing season) to 10.90 Mg C ha^−1^ year^−1^ (2017 season). These opposing temporal trends in GPP and TER are reflected in the widening of peak season monthly GPP:TER ratios, which increased from a seasonal maximum of 1.18 for 2014 to around 2.3 in 2017 (Figure 3). Monthly NEE sums for the main growth season, again reflecting an increasing (e.g. becoming more negative) trend with time since tillage (Figure 3), ranged from −0.32 Mg C ha^−1^ month^−1^ (August 2013) to −2.88 Mg C ha^−1^ month^−1^ (July 2017).

### 3.2 Yield and NEP

In the year immediately following the tillage event, yield was reduced to the point where it was uneconomical to harvest, with destructive sampling giving a predicted yield of 1.28 Mg odt ha^−1^ (Table 2). In the following years, modelled yields based on EC data and auxiliary measurements, show a gradual recovery in yields (Table 2). Data for the farm yields (average of all Miscanthus fields) is more variable but does also show an increase in yield during the first 3 years post tillage (Table 2), as expected from the increasing contribution of the tilled field to overall biomass yield at farm scale. Farm yields drop in 2017, however, this would have included the impact on yields following the drainage work conducted within Miscanthus field D (Figure 1) (Landowner, personal communication).

**TABLE 2:** Comparison of site yields (all four fields) and modelled tillage field yields.

Harvest year		Measured site yields	Modelled field yields
Start	End	Mg odt ha^−1^	Mg odt ha^−1^
05/04/2013	31/03/2014	1.28^a^	0.62
01/04/2014	31/03/2015	6.32	3.08
01/04/2015	31/03/2016	8.61	4.29
01/04/2016	31/03/2017	6.47^b^	5.69
01/04/2017	31/03/2018	7.94	6.30
Total		**29.34**	**19.98**

^a^ Based on destructive subsample of tilled field taken by Keane et al. (2019). Note while the crop was cut in March 2014, it was considered uneconomically to bale thus the above ground biomass was left on site.

^b^ Miscanthus Field D was not harvested due to extremely wet conditions, instead sub‐soiling was conducted to improve drainage. Site yields therefore excluded this field.

### 3.3 Net ecosystem production

**FIGURE 4:**
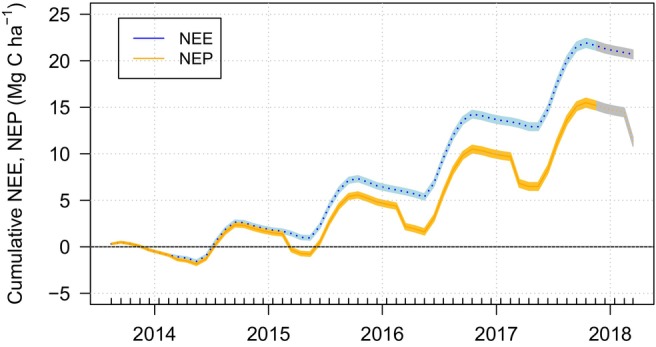
Cumulative monthly sums of net ecosystem CO_2_ exchange (NEE, blue line and shading) and cumulative net ecosystem production (NEP, orange line and shading) for a commercial Miscanthus plantation in Lincolnshire, UK. The step changes in NEP represent the removal of carbon in harvested biomass (modelled yields), which is assumed to be returned to the atmosphere during combustion for energy generation. Shaded areas for the period December 2017 to March 2017 were estimated as averages of the data collected over previous years. Shaded areas show cumulative uncertainty estimates (2*σ*) calculated using standard error accumulation principles using the root sum square of two standard deviations calculated for each 30‐min CO_2_ flux density.

NEP, with carbon uptake into the system presented as positive value, was estimated over the four harvests between August 2013 and March 2018 as 12.16 Mg C ha^−1^, after accounting for the removal of −8.52 Mg C ha^−1^ in harvested biomass based on modelled yields (Table 2; Figure 4). Cumulative NEP showing that in the year following the tillage the site was overall a small source of C, however this reversed with time with the field becoming a net carbon sink by 2015 (Figure 4). These values do not include any C offsetting that may be provided by the harvested biomass.

### 3.4 Soil C stocks

Soil C stocks in the tilled and adjacent untilled field were not significantly different at either depth increments, suggesting no direct impact of tillage (Table 3, also see Figure S5 for full depth profiles). Soil C stocks post‐tillage were also higher than pre‐tillage values (77.95 ± 2.01 Mg C ha^−1^ and 66.79 ± 3.36 Mg C ha^−1^ respectively) resulting in a positive rate of change (Table 3). Mean soil C stocks post‐tillage were also marginally higher than the paired arable field (71.56 ± 2.81 Mg C ha^−1^) equating to a rate of change over the 10 year crop life of +0.64 ± 0.95 Mg C ha^−1^ year^−1^, although this difference was not statistically significant (Table 3).

**TABLE 3:** Differences in soil C stocks and rates of changes post‐tillage for the key contrast.

Contrasts	Difference in soil C stocks (Mg C ha^−1^)		Post‐hoc contrasts	Rates of change (Mg C ha^−1^ year^−1^)
	0–30 cm	0–70 cm		0–30 cm
Zost‐tillage vs. untilled	0.35 ± 3.05^NS^	3.55 ± 4.75^NS^	*p* = 0.99	NA
Post‐tillage vs. pre‐tillage	11.16 ± 3.91^S^		*p* = 0.02	2.23 ± 0.78
Post‐tillage vs. arable	6.39 ± 3.45^NS^		*p* = 0.34	0.64 ± 0.95
Mix model, main effect of land management	*χ*_(3)_ 19.53 *p* ≤ 0.001	*χ*_(2)_ 0.74 *p* = 0.391		

*Note*: Values are based on equivalent soil mass, are given with pooled standard error, post‐hoc contrast gives the tukey test post hoc *p* values following significant main effect. Post tillage refers to the 2016 soil samples for Mis A field. Untilled refers to the 2016 samples from Mis B field. Pre‐tillage refers to the 2011 samples from Mis A field. Arable refers to the arable field representing the original land‐use on which the Miscanthus was planted following the space for time approach (REF). “S” mark significant difference. “NS” none significant differences between values, for 0–30 cm this is based on Tukey post‐hoc test of a significant main effect.

### 3.5 Ecosystem C dynamics

**FIGURE 5:**
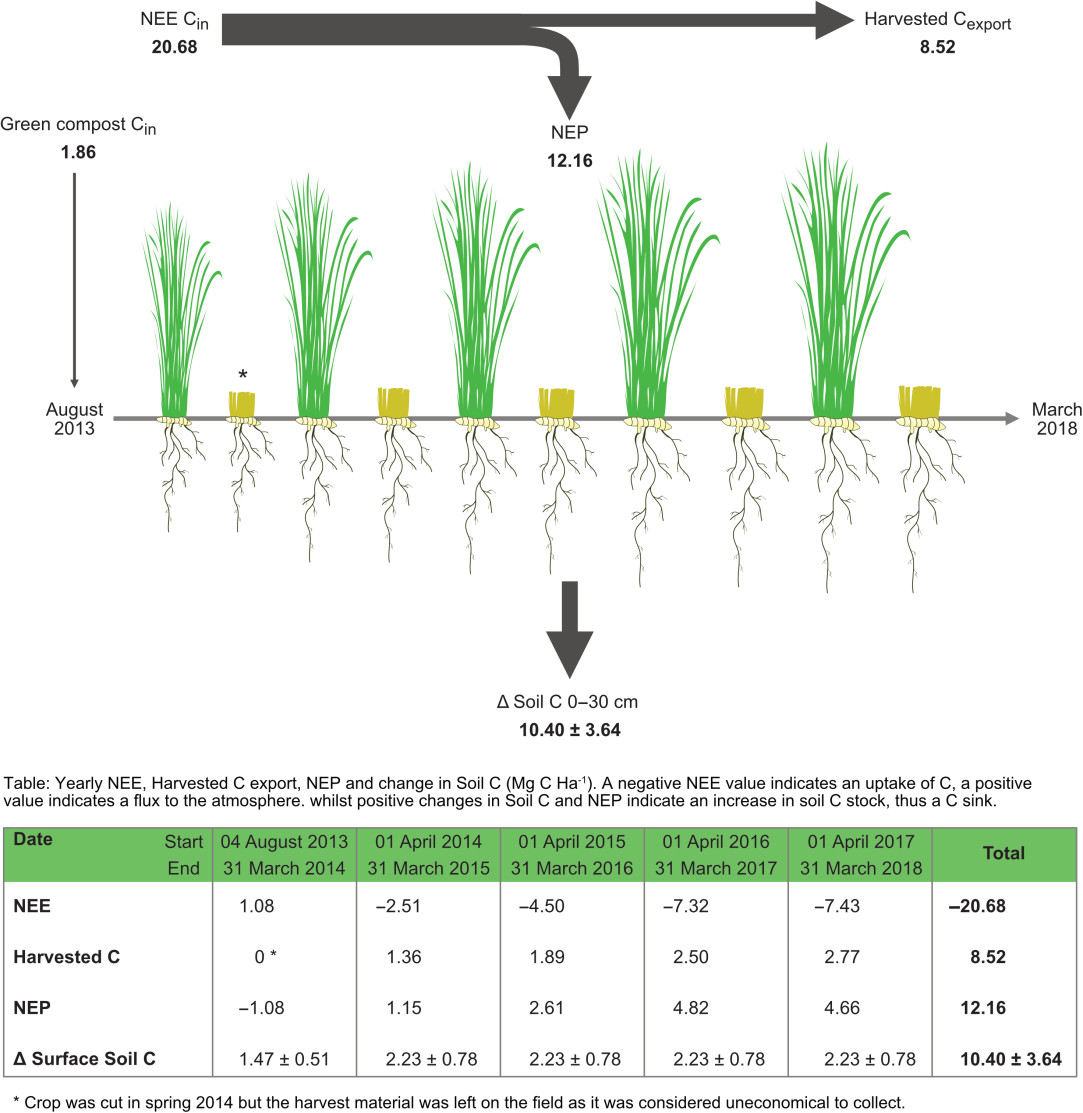
Summed C fluxes between July 2013 and March 2018 and estimated changes in soil C stock. Values are in Mg C ha^−1^ and harvested C export is based on modelled yields.

Between July 2013 and March 2018, the NEP based on modelled yields was 12.16 or 7.64 Mg C ha^−1^ based on the whole site yields, indicating an increase in ecosystem C stock within the Miscanthus field (Figure 5). Both values are within the error of the predicted increase in soil C stocks over the same time period, 10.40 ± 3.64 Mg C ha^−1^ (Figure 5), based on the soil C rates of change for the pre‐post tillage periods of 2.23 ± 0.78 Mg C ha^−1^ year^−1^. There was also an addition of 1.86 Mg C ha^−1^ with the green compost (wood waste) to the soil, the fate of which (sequester vs. respired) may not be fully captured as application occurred shortly before EC measurement commenced due to the need to accommodate field operations and dissolved organic carbon losses were not measured. The 8.52 Mg C ha^−1^ exported in the harvested biomass, was assumed to be returned to the atmosphere during combustion for energy generation.

## 4 DISCUSSION

For the first time, we have measured the impacts of remedial tillage on soil C stocks, carbon (C) fluxes and net ecosystem C balance. No tillage induced impacts on soil C stocks were detected, with no significant difference, 3 years post tillage, in the surface (0–30 cm) or deep (0–70 cm) soil C stocks between the tilled Miscanthus field (Mis A) and the paired untilled Miscanthus field (Mis B). Further‐more, in comparison to the historical samples, surface soil C stocks increased by 11.16 ± 3.91 Mg C ha^−1^ between pre (2011) and post tillage (2016) sampling, an annual rate of change of 0.64 ± 0.95 Mg C ha^−1^ year^−1^. Impacts of the tillage on the subsequent growth of the crop were, however, observed in the low NPP measured using EC and the yield data, with tilled field unlike the other Miscanthus fields at this site, being left unharvested due to the extremely low yield. The EC provided a more detailed measurement of temporal C dynamics, albeit over a slightly different time period to that represented by the soil sampling. NEE was positive in the first harvest cycle following tillage, indicative of a net loss of C stocks. Yields were also reduced to the point that it was considered uneconomical to harvest the AGB. NEE and yields demonstrated a recovery in the subsequent years, the NEP (NEE − C exported in yield) showing the field had functioned as a cumulative net C sink of 12.16 Mg C ha^−1^ by March 2018.

### 4.1 Potential drivers of changes in temporal C fluxes following tillage

Whilst both total respiration and crop production increased over the study period, changes in the GPP:TER (the ratio of GPP to respiration) suggests that respiration was a proportionately higher fraction of assimilated C in the first few years following tillage, declining with time since tillage. Alongside lower rates of photosynthesis in the year immediately after tillage, this proportionally higher release of assimilated CO_2_ via respiration relative to later years is likely a significant cause of the positive NEE fluxes in 2014, and contributed to lowering NEP values in the following 2 years. Potential reasons for elevated respiration rates after tillage include heterotrophic decomposition of Miscanthus roots and rhizomes severed during mechanical tillage, decomposition of green wood waste compost, and/or an increase in autotrophic maintenance respiration as the Miscanthus invested resources in new rhizome and root production. Studies of Miscanthus removal, where tillage is combined with herbicide application, have also reported increased respiration rates, with isotopic analysis confirming that, at least during the first few weeks, decomposition of Miscanthus roots and rhizomes contributes a significant fraction of the observed increase in C soil emissions (Drewer et al., 2016; Dufossé et al., 2014). Similarly, a longer‐term study over 2 years of the reversion of perennial switchgrass, which like Miscanthus has an extensive belowground root system, to maize cropping reported significantly increased rates of heterotrophic respiration (Moore et al., 2020). In this case, consummate measurements of below ground biomass (BGB) led the authors to conclude that the increase in respiration was driven by the relative rapid decomposition of root material (Moore et al., 2020). Rapid decomposition of BGB, in some cases in as few as 1–2 years, has also been indicated in studies of soil C stocks following Miscanthus crop removal (Dufossé et al., 2014; Martani et al., 2022; Rowe et al., 2020). Based on the AGB to BGB ratio reported in Beuch et al. (2000) prior to the tillage in 2013 there would have been approximately 5.27 Mg C ha^−1^ stored within the roots and rhizomes. Enough to make a significant contribution to the 7.78 Mg C ha^−1^ of TER observed in the first harvest cycle following reversion and the resulting lower GPP:TER. Unlike crop removal, however, remedial tillage involves lower levels of mechanical soil disturbance and no use of herbicide, thus although necrotic rhizomes were observed on the soil surface within the tilled field (author's personal observation), it is likely that only a proportion of the BGB was damaged and therefore subject to decomposition. The addition of waste wood compost may have also contributed to the increased respiration rates. Mulching with composted wood has been shown to increase respiration and microbial activity (Tiquia et al., 2002). The compost applied to the site represented an input of 1.86 Mg C ha^−1^ and whilst the rate of decay of the wood waste compost applied to the site is not known, it does seem likely that this application will have contributed to the respiration measured. Additionally, the carbon use efficiency of the crop may have been reduced following tillage, with a greater investment required for rebuilding root networks and mycorrhizal associations following tillage resulting in higher rates of respiration.

It is also not possible to rule out temporary losses of soil C following the tillage contributing to the higher respiration, with these losses compensated with later gains resulting in the absence of any observable impacts within the soil samples. Such direct loses of soil C have been proposed as a contributing factor to analogous patterns of changes in GPP:TER ratios observed in the early stages of land use change to bioenergy (McCalmont, McNamara, et al., 2017; Ní Choncubhair et al., 2017; Zenone et al., 2013). Future quantification of the relative contribution of these different potential drivers may open pathways to reduced unwanted respiration and the associated climate forcing. However, it should be remembered that even with this higher initial respiration the crop returned to a negative NEP balance within three harvest cycles. This follows the pattern reported by McCalmont, McNamara, et al. (2017) for the establishment of Miscanthus on grassland, where an initial flux of GHG emissions driven by decomposition of the previous grass crop was offset over the following years by C uptake by the Miscanthus crop.

### 4.2 Rates of change in soil C stocks

Comparison between the tilled and untilled fields suggests the tillage had no impact on the rate of change in soil C stock in either the surface soil or the deeper 0–70 cm soil profile. Within the tilled field comparison to the pre‐tilled samples from 2011 showed that surface soil (0–30 cm) C stock had increased over this time period by 11.16 ± 3.91 Mg C ha^−1^. This equates to a rate of change of 2.23 ± 0.78 Mg C ha^−1^ year^−1^. Although, the lifetime rate of change for the tilled field, calculated by comparison to the arable field, is lower at 0.64 ± 0.95 Mg C ha^−1^ year^−1^. This life time rate of change is at the upper bounds of values reported by the authors (Rowe et al., 2016) of −0.93 ± 0.74 Mg C ha^−1^ year^−1^ (*n* = 11) from a study of 11 commercial Miscanthus plantations established on arable land across the UK, including the historical samples from this site. This study however contained a number of young plantations with a mean age of 6.4 years, and as was seen at this site soil C stocks are can increase overtime (Qin et al., 2016). In a more comparable study of an 11 year old Miscanthus plantation in Germany the authors report a similar rate of change of 0.66 Mg C ha^−1^ year^−1^, for unfertilized and non‐irrigated Miscanthus (Gauder et al., 2016). The values are also within the range reported in the review by McCalmont, Hastings, et al. (2017) of −0.26 to +3.8 Mg C ha^−1^ year^−1^, and the meta analysis by Qin et al. (2016) of 0.44 to 2.23 Mg C ha^−1^ year^−1^ (*n* = 23, upper and lower quartile). Although both theses reviews includes studies using fixed depth sampling methodology, which can inflate difference (Haden et al., 2020).

The differences in rate of change observed during the different time periods, 2.23 ± 0.78 Mg C ha^−1^ year^−1^ between the sampling periods, verse 0.64 ± 0.95 Mg C ha^−1^ year^−1^ the life time of the crop, highlight the temporal soil C dynamics at this site. Soil C stocks switched from being non‐significantly lower that the arable field in 2011 to non‐significantly higher in 2016. Such reversals in rates of soil C change with time have also been observed in other studies where initial losses in soil C stock during land use change from annual to perennial systems are recovered over the life time of the crop (McCalmont, McNamara, et al., 2017). Qin et al. (2016) also notes the relationship between crop age and rates of change within their meta‐analysis, with both the magnitude and variability in values for rates of the changes reducing with crop age for a range of perennial crops (Qin et al., 2016). This emphasizes the need to consider the time period when assessing the impacts of bioenergy crops on soil C stock. Reliable detection of changes in soil C stock from soil sampling can take up 10 years due to the slow rates of change and a high levels of spatial variability (Smith, 2004), highlighting the value of long‐term studies and combining measurement approaches with soil C modelling (Smith et al., 2020).

### 4.3 Impact of tillage on crop yield and C offsetting potential

The crop yield in the first year following the remedial tillage was reduced to the point that it was considered uneconomical to harvest the AGB, with destructive sampling conducted by Keane et al. (2019) giving the yield at 1.28 ± 0.19 Mg odt ha^−1^. This results in a yield debt, and thus a reduction in the C offsetting this crop would have otherwise provided had tillage not occurred. The expectation is that tillage will replace this loss by increasing yields in the following years, however, neither the mean (2015–2018) post tillage sites (average of all fields including the tillage field) yield, 7.34 Mg odt ha^−1^ nor modelled tillage field yield, 4.86 Mg odt ha^−1^ were higher than those reported pre tillage, 7.5 Mg odt ha^−1^ (Robertson, Whitaker, et al., 2017). This is concerning, but the impacts must also be considered in the context of the alternative management options for this field. Whilst not undertaking tillage would be an alternative, the landowner was clearly motivated to take steps to improve productivity. The remaining alternative would be to replant with the current or a superior Miscanthus variety if available. This, however, would likely result in a very similar yield debt, as the newly planted Miscanthus field would take time to establish with low yields in the first 2–3 years, (Shepherd et al., 2020). Indeed the modelling of yields with this study, which showed a close match to the EC data, was based on the expected sigmoidal establishment curve for *Miscanthus* × *giganteus* (Shepherd et al., 2020), suggesting the recovery of the crop post tillage was analogous to the process of crop establishment. Remedial tillage may have advantages over the removal and re‐establishment of a new crop. New planting material is not required thereby avoiding the C and financial cost associated with the production and transport of this material. Remedial tillage also avoids the need to remove the current crop, a process that may, based on a study by Rowe et al. (2020), have significant negative impact on soil C stocks. In this reversion study by Rowe et al. (2020) the removal of Miscanthus crops and the reversion to arable cropping results in one case where soil C losses following Miscanthus removal were great enough to negate the crops lifetime C saving through fossil fuel replacement. If the similar losses would result from the removal and replanting of Miscanthus, rather the reversion to arable cropping is unclear; however, the results of this study do suggest that remedial tillage may be a more conservative method to re‐establishment and are certainly a lower cost approach. The results reported here also only cover the 4.6 years post tillage. Yearly increases in yield and NEE were still occurring suggesting the crop may not have yet reached its maximum yield. It is also as yet unknown if the tillage will have extended the lifetime of the crop. The overall impact of tillage on the C balance of the crop requires additional work to assess all potential counterfactuals, incorporating the GHG emission associated with crop tillage and replanting (machinery use, propagation of planting material etc.) which is outside of the scope of this work.

### 4.4 Ecosystem C dynamics

This work was not designed to provide a full C budget for the site; rather it took advantage of an opportunity presented by a landowners' management choices, thus not all C flows were accounted for. There were no measurements of changes in BGB, dissolved organic carbon, nor of soil methane emissions. Although in the past measurements of soil methane emissions have been conducted at this site and have been found to be negligible and often below measurable detection limits (Robertson, Whitaker, et al., 2017). There was also not complete alignment of the timing of the soil C stock and EC measurements. Despite this, there was relatively close agreement between the NEP (12.16 Mg C ha^−1^) and the predicted changes in soil C stocks over the same time period (10.38 ± 3.63 Mg C ha^−1^). Although it should be noted there is also the potential for the soil C stock to have been elevated by the addition of the 1.86 Mg C ha^−1^ within the wood compost, although only a proportion of this will have been transferred to the soil C pool.

Even if this match between the carbon fluxes and soil C stocks was not present, the overall increase in soil C stock is indicative of the transfer of atmospheric C into the soil carbon pool. Based on the measurements taken within this study, it is not known however, the pathway by which this is occurring. In non‐till systems, the transfer of atmospheric C into the soil C pools occurs primarily via root turnover, root exudates and litter inputs (Robertson, Davies, et al., 2017). The tillage would have likely resulted in at least a temporary reduction in the inputs from roots, with lower levels of AGB, also reducing litter inputs. Therefore, it is perhaps surprising that there was not a significant difference in the soil C stock between the tilled and non‐tilled Miscanthus fields. Detection of small changes in soil C stock are difficult, and can be missed due to insufficient sampling. The sampling method used here was however, designed specifically to capture both small and large‐scale spatial variability in soil C, and the depth profiles (Figure S6) show a high level of similarity in variance. An alternative explanation is that impacts on soil C stock of the tillage either did not occur or were mitigated. Tillage will have provided a pulse of potential C inputs from severed roots and rhizomes. Authors of studies on Miscanthus removal have suggested that similar, although likely larger, inputs from damaged BGB can act as a buffer, helping to maintain soil carbon stocks, resulting in the observed non‐significant changes in soil C during the first year following the reversion of Miscanthus plantation to arable cropping (Drewer et al., 2016; Dufossé et al., 2014). Understanding if such processes were active within the tilled field site would have required additional measurements of changes in belowground biomass and ideally isotopic labelling and subsequent fractionation of the soil C pools (Dondini et al., 2009). Overall however, it must not be forgotten that the primary finding of this work is that despite the tillage the tilled field remained a net C sink.

## 5 CONCLUSION

The soil C stock and EC fluxes reported in this study suggest that, at least for this site, tillage did not result in any long‐term negative impact on carbon stock, with stocks increasing and the site being a net C sink within 2 years of the tillage activity. Tillage did however initial lower crop growth and, within the time period of this study, there was no indication that tillage resulted in the increase in yields necessary to “pay back” the yield loss. The full implication of tillage will depend on crop longevity and long‐term yield impacts, although at this site the modelling also indicates that soil and climatic conditions maybe limiting yield thus studies at additional sites would be beneficial.

## AUTHOR CONTRIBUTIONS

**Rebecca Louise Rowe:** Conceptualization; data curation; formal analysis; investigation; methodology; visualization; writing – original draft. **Hollie Mercedes Cooper:** Investigation; visualization; writing – review and editing. **Astley Hastings:** Formal analysis; funding acquisition; methodology; software; writing – review and editing. **Abigail Mabey:** Formal analysis; investigation; writing – review and editing. **Aidan M. Keith:** Conceptualization; formal analysis; investigation; methodology; writing – review and editing. **Niall P. McNamara:** Conceptualization; funding acquisition; project administration; writing – review and editing. **Ross Morrison:** Conceptualization; data curation; formal analysis; investigation; methodology; software; visualization; writing – review and editing.

## CONFLICT OF INTEREST STATEMENT

The authors declare no conflicts of interest.

## Supporting information


Data S1.

## Data Availability

Eddy covariance data is available from the NERC Environmental Information Data Centre: NERC (Morrison et al., 2019). Eddy covariance measurements of carbon dioxide, energy and water fluxes at a commercial *Miscanthus* × *giganteus* plantation, Lincolnshire, UK, 2013 to 2017 (Dataset). https://doi.org/10.5285/71e5b799‐fc4d‐4a44‐8860‐a5e358c807fd. Soil C data (stocks, variance and change), and crop management data are given in the text and tables. The corresponding author (R.L.R.), welcomes any requests for additional data; however, the data on exact site location will not be available as it contains information that could compromise the privacy of the land owners.
