# Supplementary material for: Low risk management intervention: Limited impact of remedial tillage on net ecosystem carbon balance at a commercial Miscanthus plantation
Source: Glob Change Biol Bioenergy. 2023 Dec 8;16(1):e13114. doi: 10.1111/gcbb.13114 (PMC11073546; doi:10.1111/gcbb.13114)
Supplement: Supplementary file 1 — Data S1. [file GCBB-16-0-s001.docx]

**Supplementary Material**

**Analysis of gaps within the Miscanthus crops**

No direct assessment of canopy cover was made prior to the tillage thus to assess the level of canopy cover historical aerial images were accessed (Google Earth) from 7/2008 (S1a) and gaps manually measured. Large areas of poor establishment (with little or no canopy cover) covered 11.88 % of the crop area. Assessment of sub plots for present of smaller open areas suggested smaller gaps covered an additional 10- 15%, totalling 25%. Images from post tillage 8/2020 show the absent of any large canopy gaps (figure S1B).


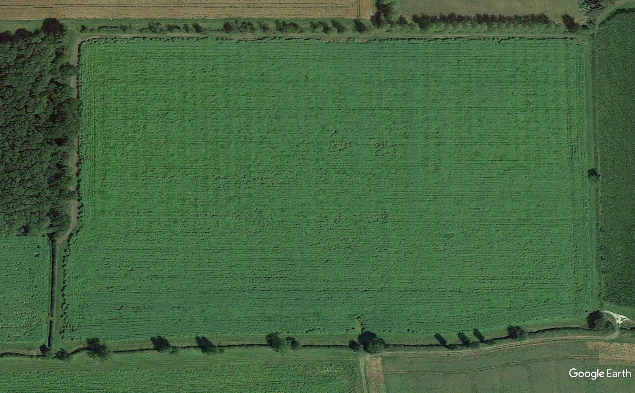


B: Post tillage 8/7/2020


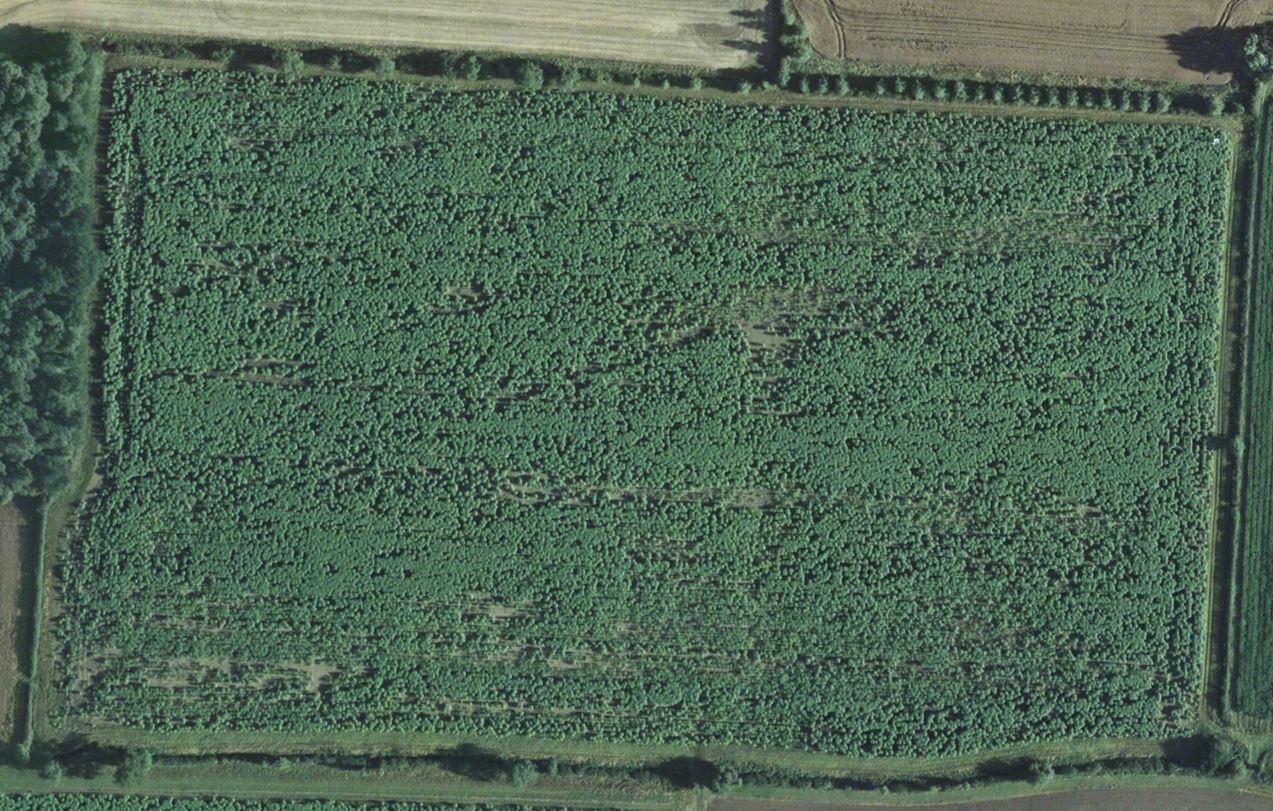


A: Pre tillage 7/2008

Figure S1. Satellite images (Google Earth) per and post tillage showing the present of open areas with the crop.

**EC footprints**

2015

2014


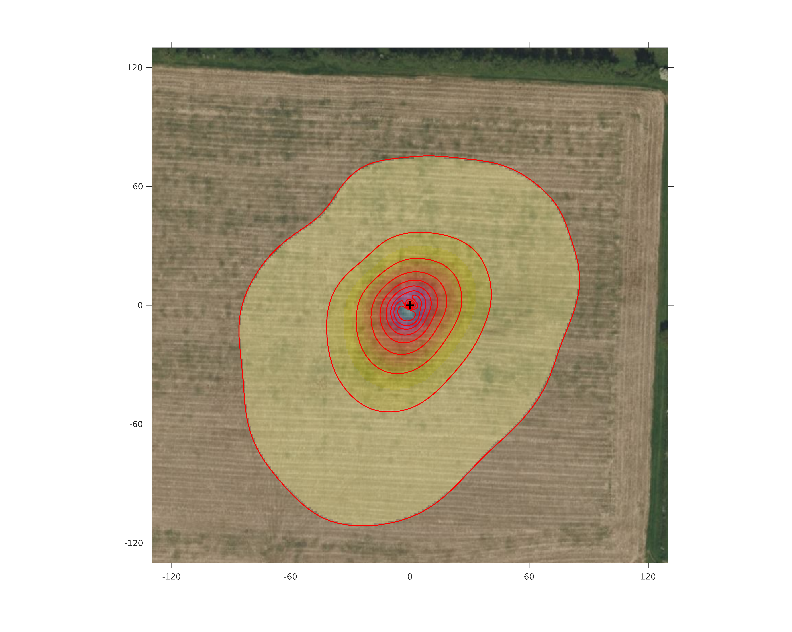

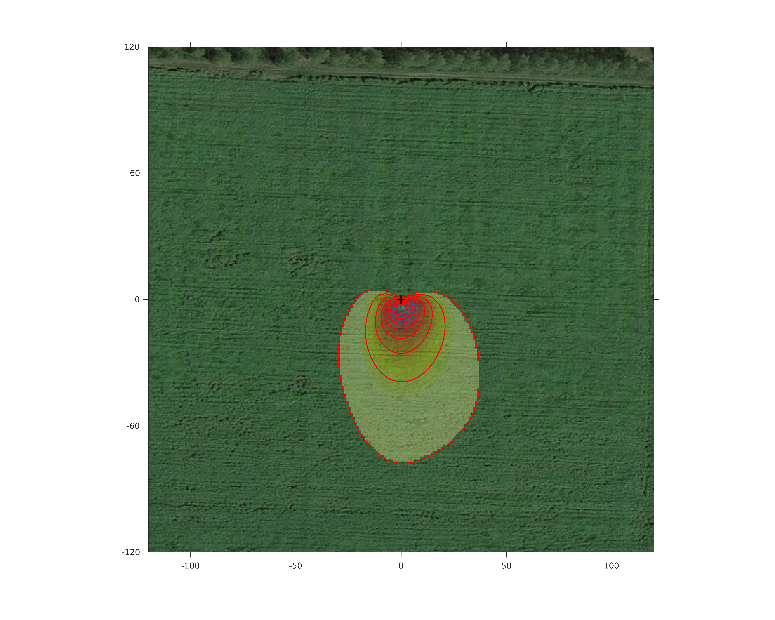

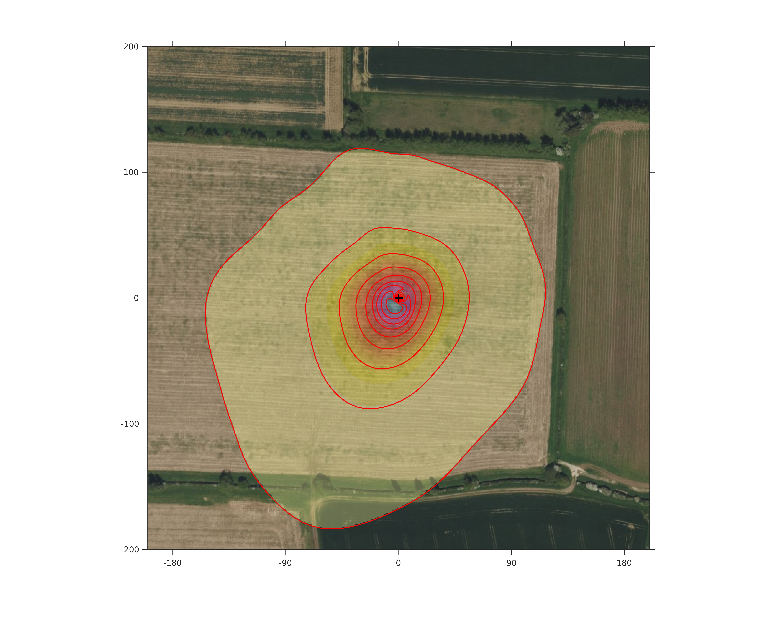

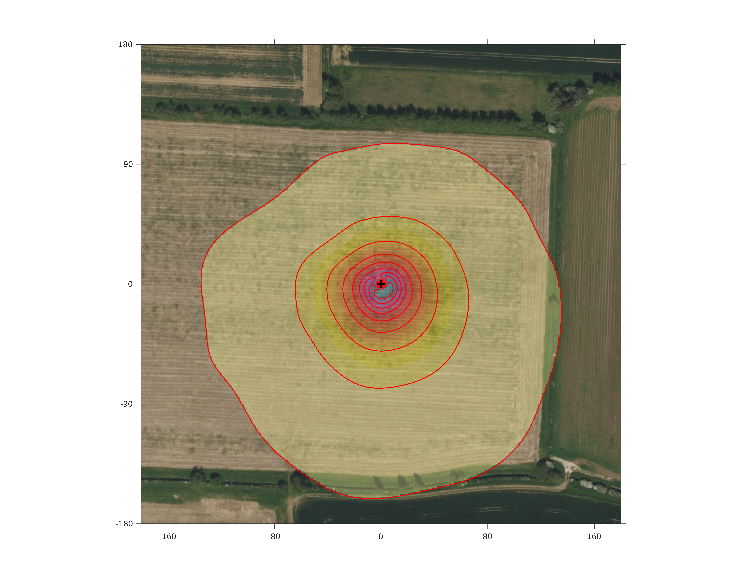

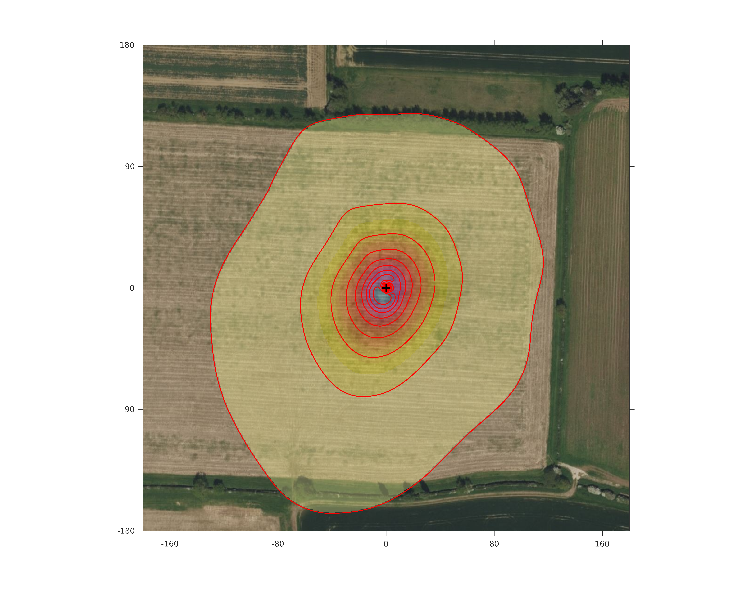


2013

2017

2016

Figure S2: Results of two-dimensional footprint analysis on the dataset using the FFP tool of Kljun et al.,2015. The flux mast is shown as red dot, contour lines show contribution of the footprint to the flux measurement in steps of 10 %. The resulting footprint climatology shows that the majority of the 90% contour was within the field most of the time, except for certain wind directions. An 80 % cut-off was used, ensuring that all measurements were representative of the cropped area.

**Modelling of yields, comparisons to EC and site yields**

To explore model fit comparisons were made between model yields and the site average yield on a per ha basses (figure S3) and between yield based on estimates made for the NPP data provided by the EC and the modelled yields (figure S4).


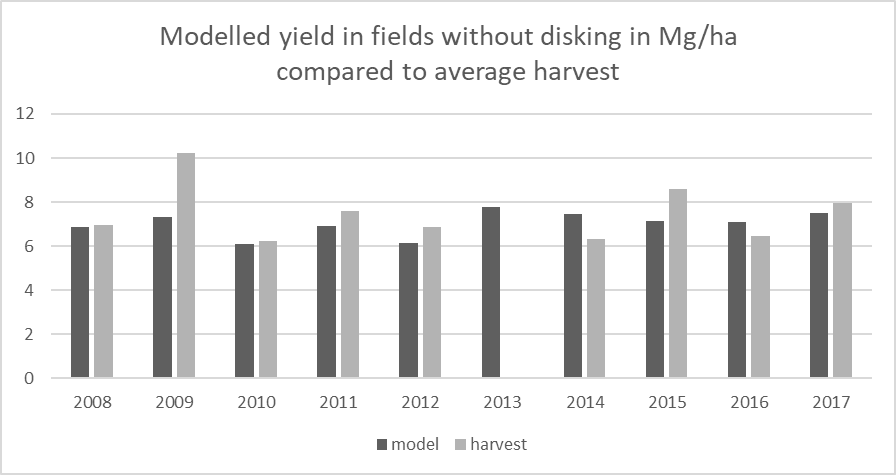


Figure S3: Comparison of modelled yields for fields without disking and the site average yields including all fields. It should be noted that year refers to growth increment in the growth year and harvest is made the following spring this 2008 refers to growth period March 2008 – March 2009


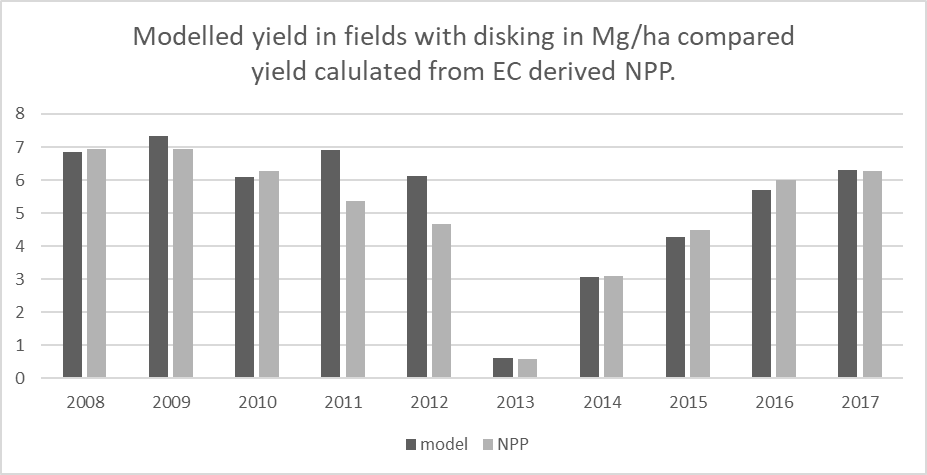


Figure S4: Comparison of modelled yield and yield estimates based on NPP values form EC system. . It should be noted that year refers to growth increment in the growth year and harvest is made the following spring

**Soil sampling methodology**

Soil in each field was sampled using a hierarchical design in order to capture variability across different spatial scales (Rowe et al., 2016). Five sampling plots per field were randomly selected from intersections of a grid overlaid on a map of the cropped area of field (Figure S4). The resolution of the grid was adjusted to ensure that there were a minimum of 50 grid intersections, with the resolution of the grid not being less than 5 m. A 20 m perimeter buffer was employed to reduce potential edge effects.

Within each of the five sampling plots, three within-plot soil cores were taken using a split-tube soil sampler (Eijkelkamp Agrisearch Equipment BV, Giesbeek, The Netherlands) with an inner diameter of 4.8 cm to a depth of 30 cm. The first core was taken at the grid intersect, with two further cores taken at distances of 1 m and 1.5 m in random compass directions from the intersect. At three randomly selected locations, the 30 cm coring was extended to 1 m by using a window sampler system with a 4.4 cm cutting diameter (Eijkelkamp Agrisearch Equipment BV, Giesbeek, The Netherlands). The window sampler being placed with the hole generated by the 30 cm sampling immediately after the removal of the 30 cm core, to extend the sampling to 1 m in depth. This combination of coring methods is used to minimize compression of the surface soil, which is more problematic with mechanically driven window sampling systems.


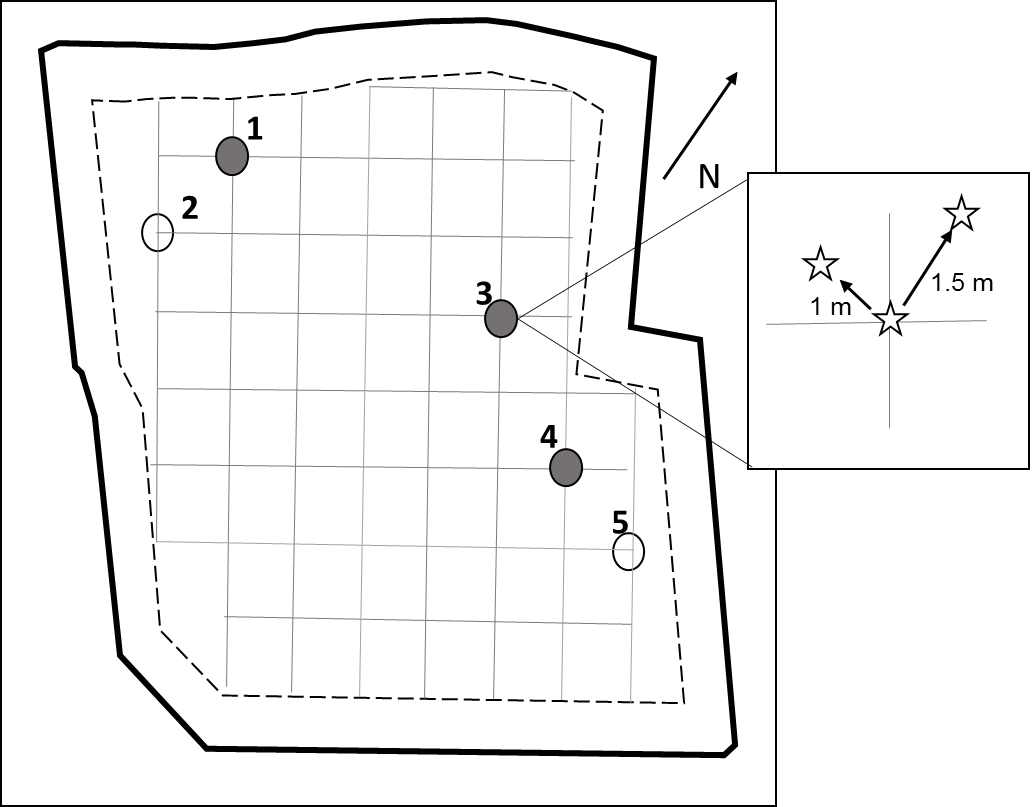


**Figure S5: Arable field showing the experimental design for the selection and layout of sampling points within each field.** The solid black line shows the extent of the cropped area excluding headlands. The dotted line shows the 20 m buffer around the cropped area and the solid grey lines the overlaid grid. Grid intersections are numbered from top right to bottom left (not shown) and a random number generator (https://www.random.org) is then used to select 5 sampling points (marked 1 -5). White circles show location of 30 cm only coring, grey filled circles show locations where coring is extended to 1 m. Insert shows the three within plot coring locations, the first positioned on the grid intersection, the remaining two located at 1 m and 1.5 m distance in random compass directions.

**Depth Profiles:**


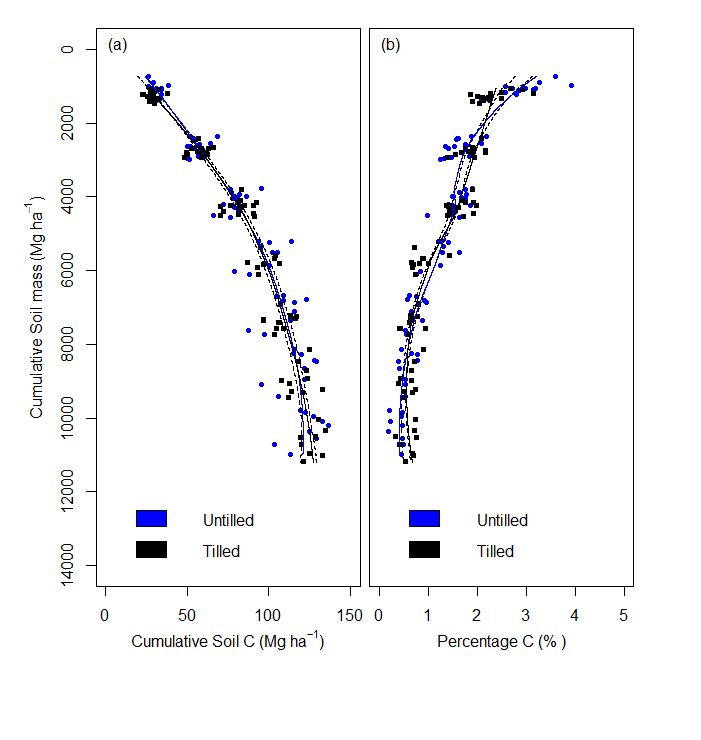


Fig S6: Bootstrapped Loess Regression plots of cumulative soil mass versus soil carbon stock for the tilled and none tilled Miscanthus fields see (A.M. Keith, Henrys, Rowe, & McNamara, 2016) for details on method. This method allows comparison of soil C stock and % C across the full depth profile. Confidence interval is represented by the area between the dotted lines; this is the null hypothesis of no differences between the land uses; solid lines represent Loess regression of each land use. As loess regression line Soil C stocks for both the fields fall within the confidence interval, the null hypothesis is accepted. % C regression show some indication of impacts in the surface samples (0- 10 and 0- 20 cm) with a reduction in the slope of the line in the tilled field, this is consistent with tillage induced mixing of the surface soil and a reduction is soil C stratification with depth.
